# Evolving outcomes of extracorporeal membrane oxygenation support for severe COVID-19 ARDS in Sorbonne hospitals, Paris

**DOI:** 10.1186/s13054-021-03780-6

**Published:** 2021-10-09

**Authors:** Matthieu Schmidt, Elise Langouet, David Hajage, Sarah Aissi James, Juliette Chommeloux, Nicolas Bréchot, Petra Barhoum, Lucie Lefèvre, Antoine Troger, Marc Pineton de Chambrun, Guillaume Hékimian, Charles-Edouard Luyt, Martin Dres, Jean-Michel Constantin, Muriel Fartoukh, Pascal Leprince, Guillaume Lebreton, Alain Combes, for the GRC RESPIRE Sorbonne Université

**Affiliations:** 1 Inserm, UMRS_1166-ICAN, Institute of Cardiometabolism and Nutrition, Sorbonne Université, Paris, France; 2 Service de Médecine Intensive-Réanimation, iCAN, Institut de Cardiologie, Assistance Publique–Hôpitaux de Paris (APHP), Sorbonne Université Hôpital Pitié–Salpêtrière, 47, Bd de L’Hôpital, 75651 Paris Cedex 13, France; 3 GRC 30, RESPIRE, APHP, Hôpital Pitié-Salpêtrière, Sorbonne Université, Paris, France; 4 INSERM, Institut Pierre-Louis d’Epidémiologie Et de Santé Publique, APHP, Hôpitaux Universitaires Pitié–Salpêtrière Charles Foix, Département de Santé Publique, Centre de Pharmacoépidémiologie (Cephepi), Sorbonne Université, CIC-1421 Paris, France; 5 APHP, Hôpital Pitié–Salpêtrière, Service de Pneumologie, Médecine Intensive–Réanimation (Département “R3S”), Sorbonne Université, Paris, France; 6 Inserm, UMRS_1158 Neurophysiologie Respiratoire Expérimentale Et Clinique, Sorbonne Université, Paris, France; 7 GRC 29, APHP, DMU DREAM, Department of Anaesthesiology and Critical Care, Pitié–Salpêtrière Hospital, Sorbonne University, Paris, France; 8 APHP, Sorbonne Université, Hôpital Tenon, Service de Médecine Intensive Réanimation, Sorbonne Université, Paris, France; 9 Service de Chirurgie Cardiaque, Institut de Cardiologie, APHP, Paris, Sorbonne France

**Keywords:** Extracorporeal membrane oxygenation, Venovenous ECMO, Acute respiratory distress syndrome (ARDS), COVID-19, SARS-CoV-2, Outcomes

## Abstract

**Background:**

Extracorporeal membrane oxygenation (ECMO) was frequently used to treat patients with severe coronavirus disease-2019 (COVID-19)-associated acute respiratory distress (ARDS) during the initial outbreak. Care of COVID-19 patients evolved markedly during the second part of 2020. Our objective was to compare the characteristics and outcomes of patients who received ECMO for severe COVID-19 ARDS before or after July 1, 2020.

**Methods:**

We included consecutive adults diagnosed with COVID-19 in Paris–Sorbonne University Hospital Network ICUs, who received ECMO for severe ARDS until January 28, 2021. Characteristics and survival probabilities over time were estimated during the first and second waves. Pre-ECMO risk factors predicting 90-day mortality were assessed using multivariate Cox regression.

**Results:**

Characteristics of the 88 and 71 patients admitted, respectively, before and after July 1, 2020, were comparable except for older age, more frequent use of dexamethasone (18% vs. 82%), high-flow nasal oxygenation (19% vs. 82%) and/or non-invasive ventilation (7% vs. 37%) after July 1. Respective estimated probabilities (95% confidence intervals) of 90-day mortality were 36% (27–47%) and 48% (37–60%) during the first and the second periods. After adjusting for confounders, probability of 90-day mortality was significantly higher for patients treated after July 1 (HR 2.27, 95% CI 1.02–5.07). ECMO-related complications did not differ between study periods.

**Conclusions:**

90-day mortality of ECMO-supported COVID-19–ARDS patients increased significantly after July 1, 2020, and was no longer comparable to that of non-COVID ECMO-treated patients. Failure of prolonged non-invasive oxygenation strategies before intubation and increased lung damage may partly explain this outcome.

**Supplementary Information:**

The online version contains supplementary material available at 10.1186/s13054-021-03780-6.

## Introduction

Extracorporeal membrane oxygenation (ECMO) was frequently used to treat coronavirus disease-2019 (COVID-19) patients with severe acute respiratory distress syndrome (ARDS) during the initial outbreak from January to June 2020 [1–4]. High-volume ECMO centers and large ECMO networks reported similar survival rates for these patients compared to ECMO-supported patients with non-COVID-associated ARDS [5–7].

Management of COVID-19 patients evolved over the following months, as knowledge of the disease improved with the publication of landmark randomized trials. In June 2020, RECOVERY-trial results showed that dexamethasone (6 mg/day for 10 days) significantly reduced mortality compared to usual care for patients receiving either invasive mechanical ventilation or oxygen alone. That dexamethasone regimen was recommended by the World Health Organization and largely prescribed to COVID-19 patients. High-flow oxygen through a nasal cannula (HFNO) or noninvasive ventilation (NIV) was also shown to lower COVID-19 patients’ need for invasive mechanical ventilation and was recommended in national and international guidelines.

Because the care of COVID-19 patients evolved during the second part of 2020, we compared the characteristics and outcomes of patients who received ECMO support before and after July 1, 2020, for laboratory-confirmed severe acute respiratory distress syndrome (ARDS)-associated coronavirus-2 (SARS-CoV-2) infection in Paris–Sorbonne Hospitals.

## Methods

### Study settings

All consecutive adults, with laboratory-confirmed SARS-Cov-2 infection admitted to Paris–Sorbonne University Hospital Network ICUs, who received venoarterial (VA)- or venovenous (VV)-ECMO for severe ARDS from March 8, 2020, to January 28, 2021, were included, and provided 90-day survival status. The characteristics and outcomes of the first 83 cohort patients were reported previously [2]. We chose to split that cohort into two periods, before and after July 1, 2020, respectively. That date corresponds to the publication of the RECOVERY trial [8], and the transition period between the first and second COVID-19 waves in France. ECMO support was provided in four ICUs at Pitié–Salpêtrière and Tenon Hospitals before July 1, and in one Pitié–Salpetrière Hospital ICU thereafter. The Sorbonne University Ethics Committee approved the study protocol (CER-SU-2020–46).

### ECMO indications and organization

ECMO indications for COVID-19 patients are detailed elsewhere [2, 4] and did not change throughout the study period. Briefly, indication(s) for ECMO implantation were centralized and evaluated in staff meetings, including at least two intensivists. Severe ARDS patients eligible for ECMO had to fulfill EOLIA trial respiratory severity criteria [5], with pre-ECMO use of neuromuscular-blocking agents and prone-positioning strongly recommended. Similarly, ECMO contraindications were still age > 70 years, severe comorbidities, cardiac arrest, refractory multiorgan failure or Simplified Acute Physiology Score (SAPS) II [9] > 90, irreversible neurological injury and/or invasive mechanical ventilation for > 10 days. Once the indication was retained, the Pitié–Salpêtrière Mobile ECMO Retrieval Team, comprising a cardiovascular surgeon and a perfusionist, was sent at the patient’s bedside for ECMO cannulation and retrieval to our department, as described previously [10, 11].

### Management of ECMO for COVID-19

Throughout the study period, femoral–jugular percutaneous cannulation under ultrasonography guidance with a large drainage femoral cannula (25–29Fr) was strongly recommended for VV-ECMO. Pump speed was adjusted to obtain > 90% arterial oxygen saturation. Optimal cannula positioning was verified by ultrasonography and chest X-ray. Because of frequent thromboembolic events on-ECMO, including massive pulmonary embolism [12], and based on our early experience [2], the targeted activated partial thromboplastin time (aPTT) for VV-ECMO anticoagulation with unfractionated heparin was 60–75 s or anti-Xa activity 0.3–0.5 IU/mL. The hemoglobin threshold for red-cell transfusion was 7–8 g/dL and platelet transfusions were discouraged except for severe thrombocytopenia (< 50 G/L) or thrombocytopenia < 100 G/L with bleeding. To enhance protection against ventilator-induced lung injury, ultra-protective lung ventilation on-ECMO was recommended [6], and early prone-positioning on-ECMO was encouraged in the absence of hemodynamic instability and contraindications [13]. Based on the first descriptions [1, 2, 14] of very long ECMO runs and mechanical ventilation of COVID-19 patients, our team decided to decrease on-ECMO neuromuscular blocking-agent use for these patients. Patients were assessed daily for possible ECMO-weaning using the EOLIA clinical and physiological criteria [5, 15]. On-ECMO tracheostomy was considered after having identified, before the procedure, that the patient would likely tolerate decreased sedation. All tracheostomy decisions were made after discussion within the medical team of the balance between risks and benefits of that procedure on-ECMO [16]. When VA-ECMO was initiated, the ipsilateral limb received percutaneous cannulation via an anterograde perfusion catheter.

### Data collection

Our first-wave, ECMO-treated COVID-19 patients’ data were reported previously [2]. Briefly, they included pre-ECMO demographic information; severity scores (i.e., SAPS II [9]; Sequential Organ-Failure Assessment (SOFA) score [17]; Respiratory Extracorporeal Membrane Oxygenation Survival Prediction (RESP) score [18]); rescue therapies; pre-ECMO ventilatory mechanics, including driving pressure (ΔP), mechanical power [19] and ventilatory ratio [20]; arterial blood-gas parameters, and routine laboratory values. Because we expected that pre-intubation management had changed over the study period, HFNO and NIV use and their durations were also noted.

Mechanical ventilation settings, arterial blood gases, adjuvant therapies on-ECMO and ECMO-related complications were recorded daily from day 1 to 7, then every 7 days until ECMO-day 60, ECMO-weaning or death, whichever occurred first. Major bleeding was defined as the need for ≥ 2 units of packed red blood cells for an obvious hemorrhagic event, necessitating a surgical or interventional procedure, an intracerebral hemorrhage or being fatal, while massive hemolysis was defined as plasma-free hemoglobin > 500 mg/L associated with clinical signs of hemolysis.

Lastly, specific COVID-19 treatments were recorded. We specified whether dexamethasone (6 mg/day) was initiated before or during ECMO, and if the patient received high-dose corticosteroids, defined as > 1 mg/kg/day of prednisone or equivalent since ARDS onset, during the ECMO run [21].

### Outcomes

Patient outcomes on days 28, 40, 50, 60, 70, 80 or 90 post-ECMO implantation included the following endpoints: on-ECMO, in-ICU and weaned-off ECMO, alive and out of ICU or died. The time spent in each of those four states until the specified days were also calculated. Other outcomes included ICU- and ECMO-related complications.

### Statistical analyses

Patient characteristics are expressed as *n* (%) for categorical variables or median (interquartile range, IQR) for continuous parameters, as appropriate.

To describe patients’ in-ICU trajectories over time, a multi-state model was used as in our previous study [2, 22]. Briefly, this framework considers that a patient can go through different states during follow-up. Herein, the starting time was the ECMO-initiation day, making on-ECMO the initial state for all patients, potentially followed by two intermediate states: in-ICU & weaned-off ECMO or alive & out of the ICU. Because patients could die at any time during follow-up, either in-ICU or after discharge, the death is the only final absorbing state (the final state that a patient can enter but once entered cannot be left). In this four-state model (Additional file 1), each box represents a state and each arrow represents possible transitions from one state to another.

After assessing patient status, participants who did not reach the final absorbing state were right-censored. A Cox model stratified on each possible transition state was fitted to estimate transition (from one state to another) and state-occupation (for each of the four states) probabilities over time; the percentages of patients occupying each possible state were represented simultaneously over time with a stacked probability plot and reported with their 95% confidence interval (CI) on days 28, 40, 50, 60, 70, 80 and 90 post-ECMO initiation. Another figure (Additional file 2) individually displays all possible transition probabilities from one state to another over time. Mean state-occupation times (i.e., the expected length of stay in each possible state of the multi-state model) were also reported at the same times. Finally, median on-ECMO duration and length of ICU stay were established. These analyses were computed separately for the first and second waves.

Pre-ECMO risk factors for 90-day mortality were assessed for the entire cohort using univariate and multivariate Cox regression models. The variables included in the multivariate model were defined a priori, without any variable selection. Multiple imputations were used to replace missing values when appropriate. Briefly, 10 copies of the dataset were created with the missing values replaced by imputed values, based on observed data including participants’ outcomes and pre-ECMO characteristics. Each dataset was then analyzed and the results from each dataset were pooled into a final result applying Rubin’s rule [23]. Hazard ratios and their 95% CIs were estimated.

Finally, adjusted Kaplan–Meier probabilities of survival were estimated from the multivariate Cox regression model. Each subject’s survival probability over time was estimated from the model, first considering all subjects included during the first wave, and second considering all subjects included during the second wave. Then, the survival probabilities were averaged across all individuals. Finally, unadjusted and adjusted Kaplan–Meier curves were plotted on the same figure.

All the analyses were computed at a two-sided α level of 5% with R software, version 4.0.3.

## Results

### Pre-ECMO patient characteristics

Eighty-eight patients were admitted before July 1, 2020, and 71 thereafter (Table 1). Briefly, patients admitted after July 1 were significantly older, while the numbers and types of failing organs at ECMO cannulation were comparable. The first COVID-19-symptoms-to-intubation interval was longer during the second period. Respective before and after HFNO (19% vs. 82%) and/or NIV (7% vs. 37%) use and their durations before intubation were significantly higher after July 1, while the intubation-to-ECMO interval was comparable for the two periods. At ECMO cannulation, ventilation parameters, respiratory mechanics and blood gases did not differ between groups. More than 90% of the patients had received neuromuscular blocking agents and undergone prone-positioning before ECMO during both periods, while inhaled nitric oxide or prostacyclin (35% vs. 52%, *p* = 0.032) and dexamethasone (18% vs. 82%, *p* < 0.001) were used significantly more frequently after July 1. Notably, d-dimer concentrations were significantly lower in patients admitted during the second period.

**Table 1:** Pre-ECMO characteristics according to ICU admission before or after July 1, 2020

Characteristic	All (*N* = 159)	ICU admission		*p*
		Before July 1 (*N* = 88)	After July 1 (*N* = 71)	
Age, years	51 (43–58)	49 (41–56)	54 (49–60)	0.002
Male	114 (72)	64 (73)	50 (70)	0.784
Body mass index, kg/cm^2^	30.8 (27.7–35.1)	30.3 (27.7–34.0)	31.0 (27.2–37.0)	0.513
SAPS II	55 (39–64)	46 (30–57)	61 (53–68)	< 0.001
RESP score	4 (2–5)	4 (2–5)	3 (2–4)	0.020
Total SOFA score	11 (9–13)	11 (9–13)	11 (8–13)	0.599
Renal component ≥ 3	24 (15)	15 (17)	9 (13)	0.795
Cardiovascular component ≥ 3	79 (50)	44 (50)	35 (49)	0.752
Hematological component ≥ 3	4 (3)	2 (2)	2 (3)	0.556
Comorbidities				
Hypertension	64 (40)	34 (39)	30 (42)	0.644
Diabetes	54 (34)	38 (43)	26 (37)	0.525
Chronic respiratory disease^a^	24 (15)	10 (11)	14 (20)	0.143
Immunocompromised^b^	9 (6)	3 (3)	6 (8)	0.189
Time from				
First symptoms to ICU admission, days	7 (5–10)	7 (5–10)	8 (5–11)	0.452
First symptoms to intubation, days	10 (7–13)	8 (6–11)	11 (8–17)	< 0.001
ICU admission to intubation, days	1 (0–3)	0 (0–1)	3 (1–8)	< 0.001
ICU admission to ECMO, days	7 (3–9)	6 (3–7)	9 (4–12)	< 0.001
Intubation to ECMO, days	4 (1–7)	4 (3–6)	3 (1–7)	0.115
MERT retrieval on-ECMO from another hospital	135 (85)	66 (75)	69 (97)	< 0.001
High-flow oxygen before intubation	75 (47)	17 (19)	58 (82)	< 0.001
Duration, days	4 (1–7)	2 (0–4)	5 (1–8)	0.009
Non-invasive ventilation before intubation	32 (20)	6 (7)	26 (37)	< 0.001
Duration, days	3 (1–6)	2 (3–4)	3 (1–8)	0.575
Volume-assist control ventilation	156 (98)	88 (100)	68 (96)	0.094
Ventilation parameters				
FiO_2_	100 (100–100)	100 (100–100)	100 (100–100)	0.359
PEEP, cmH_2_O	12 (10–14)	13 (12–14)	12 (10–14)	0.019
Tidal volume, mL/kg PBW	6.0 (5.6–6.4)	6.0 (5.7–6.4)	6.1 (5.5–6.3)	0.661
Respiratory rate, breaths/min	30 (26–30)	30 (28–30)	30 (26–30)	0.519
Plateau pressure, cmH_2_O	31 (29–32)	31 (29–33)	30 (30–32)	0.717
Driving pressure, cmH_2_O ^c^	19 (16–21)	18 (16–20)	19 (16–22)	0.289
Static compliance, mL/cmH_2_O	21.5 (17.5–26.4)	22.1 (18.1–26.5)	20.8 (17.0–23.9)	0.148
Mechanical power, J/min ^d^	23.8 (20.2–27.1)	24.9 (21.9–27.2)	21.4 (19.2–26.4)	0.055
Ventilatory ratio	2.7 (2.2–3.1)	2.8 (2.3–3.3)	2.5 (2.0–2.9)	0.046
Last blood-gas values pre-ECMO				
pH	7.32 (7.25–7.39)	7.32 (7.24–7.38)	7.33 (7.26–7.41)	0.143
PaO_2_/FiO_2_	60 (54–69)	60 (54–68)	60 (54–74)	0.601
PaCO_2_, mmHg	56 (49–65)	57 (50–67)	53 (48–63)	0.156
PaO_2_, mmHg	63 (54–70)	64 (54–71)	63 (55–70)	0.857
Plasma bicarbonate, mmol/L	27 (23–32)	27 (24–32)	27 (23–33)	0.705
Arterial lactate, mmol/L	1.6 (1.3–2.1)	1.6 (1.4–2.0)	1.6 (1.3–2.4)	0.858
Laboratory values				
White-cell count, G/L	13.0 (9.5–18.0)	13.1 (10.0–17.1)	12.9 (8.6–18.9)	0.872
Lymphocytes, G/L	0.85 (0.50–1.33)	0.96 (0.55–1.46)	0.80 (0.50–1.14)	0.328
Dexamethasone (6 mg/day) started pre-ECMO	74 (47)	16 (18)	58 (82)	< 0.001
Rescue therapy pre-ECMO				
Any	157 (99)	86 (98)	71 (100)	0.503
Neuromuscular blockade	150 (94)	83 (94)	67 (94)	1.000
Prone positioning	146 (92)	82 (93)	64 (90)	0.487
Inhaled nitric oxide or prostacyclin	68 (43)	31 (35)	37 (52)	0.032
High-dose corticosteroids	10 (6)	6 (7)	4 (6)	1.000
Almitrine	1 (0.6)	1 (1)	0 (0)	1.000
Renal replacement therapy	5 (3)	4 (5)	1 (1)	0.381
Cardiac arrest	5 (3)	3 (3)	2 (3)	1.000

Values are expressed as median (interquartile range) or number (%)

*ECMO* extracorporeal membrane oxygenation, *ICU* intensive care unit, *MERT* Mobile ECMO Retrieval Team, *PaO*_*2*_*/FiO*_*2*_ ratio of the partial pressure of arterial oxygen to the fraction of inspired oxygen, *PEEP* positive end-expiratory pressure, *PBW* predicted body weight, *PaCO*_*2*_ partial pressure of arterial carbon dioxide, *RESP* Respiratory Extracorporeal Membrane Oxygenation Survival Prediction, *SaO*_*2*_ arterial oxygen saturation, SAPS Simplified Acute Physiology Score, *SOFA* Sequential Organ-Function Assessment

^a^Defined as chronic obstructive pulmonary disease or asthma

^b^Defined as hematological malignancies, active solid tumor or having received specific anti-tumor treatment within 1 year, solid-organ transplant or human immunodeficiency virus-infected, long-term corticosteroids or immunosuppressants

^c^Defined as plateau pressure minus PEEP

^d^Mechanical power (J/min) = 0·098 × tidal volume × respiratory rate × peak pressure – 1/2 × driving pressure. If not specified, peak pressure was considered equal to plateau pressure

### On-ECMO patient management

ECMO management during the two periods is described in Table 2 and Additional file 3. During the second period, more patients received airway pressure-release ventilation/bilevel-mode ventilation and two remained non-intubated, awake and on HFNO. Ventilation parameters were comparable during the two periods, with major decreases of driving pressure, static compliance, mechanical power and ventilatory ratio on ECMO-day 1. More than 80% of the patients were prone-positioned during ECMO, with comparable numbers of sessions during both periods, while a significantly lower percentage of patients received continuous neuromuscular blockade during the second period (94% vs. 27%, *p* < 0.001). After July 1, 60 (85%) patients received dexamethasone during the first 15 days on-ECMO and a significantly higher percentage of patients (15% vs. 37%, *p* = 0.001) received high-dose corticosteroids for non-resolving ARDS [21].

**Table 2:** Characteristics and complications during ECMO according to ICU admission before or after July 1, 2020

Characteristic	All (*N* = 159)	ICU admission		*p*
		Before July 1 (*n* = 88)	After July 1 (*n* = 71)	
*ECMO-day 1*				
Type of ECMO support				0.846
Femoral–jugular VV	150 (94)	81 (92)	69 (97)	
Femoral–femoral VV	4 (3)	3 (3)	1 (1)	
Ventilation mode				< 0.001
APRV/bilevel	144 (91)	75 (85)	69 (97)	
Volume-assist control ventilation	13 (8)	13 (15)	0 (0)	
High-flow oxygen	2 (1)	0 (0)	2 (3)	
Ventilation parameters				
Minute ventilation, L/min	3.8 (2.4–6.2)	3.5 (2.4–6.1)	4.6 (2.6–6.2)	0.455
Driving pressure, cmH_2_O	12 (12–14)	12 (12–14)	12 (12–14)	0.623
Compliance, mL/cmH_2_O	13.5 (9.5–22.1)	13.2 (9.0–20.1)	13.9 (10.0–23.3)	0.120
Mechanical power, J/min	7.2 (4.3–12.1)	6.4 (4.1–11.3)	8.1 (5.1–12.3)	0.220
Ventilatory ratio	0.76 (0.51–1.14)	0.72 (0.44–1.09)	0.77 (0.56–1.17)	0.183
Laboratory values				
Platelet, × 10^3^/mm^3^	247 (177–317)	237 (177–310)	266 (179–333)	0.320
Hemoglobin, g/dL	9.4 (8.1–10.7)	9.1 (8.0–10.3)	10.0 (8.5–11.0)	0.023
Fibrinogen, mg/L	7.1 (5.7–8.2)	6.7 (5.7–8.1)	7.1 (5.6–8.2)	0.711
d–Dimers, ng/L	4905 (2020–17,340)	5935 (2320–18,710)	2485 (1727–3655)	0.020
aPTT ratio	1.5 (1.2–1.7)	1.35 (1.16–1.6)	1.4 (1.2–1.6)	0.011
*During the ECMO run*				
aPTT ratio ECMO-day 2	1.5 (1.2–1.7)	1.5 (1.3–1.9)	1.4 (1.2–1.6)	0.011
aPTT ratio ECMO-day 3	1.7 (1.3–2.2)	1.8 (1.4–2.5)	1.5 (1.2–2.0)	0.012
Adjuvant therapies on ECMO				
Continuous neuromuscular blockers	102 (64)	83 (94)	19 (27)	< 0.001
Prone positioning	131 (82)	71 (81)	60 (85)	0.529
No. of sessions on-ECMO	2 (1–3)	2 (1–3)	2 (1–4)	0.121
Nitric oxide or prostacyclin	5 (3)	5 (6)	0 (0)	0.066
High-dose corticosteroids	39 (25)	13 (15)	26 (37)	0.001
Renal replacement therapy	63 (40)	39 (44)	24 (34)	0.178
Tracheostomy	47 (30)	26 (30)	21 (30)	0.996
Received COVID-19 specific treatment				
Remdesivir	17 (11)	8 (9)	9 (13)	0.467
Lopinavir/ritonavir	20 (13)	20 (23)	0 (0)	< 0.001
Tocilizumab	9 (6)	8 (9)	1 (1)	0.042
Dexamethasone, ≤ 6 mg/day 1^st^ 15 days	73 (46)	13 (15)	60 (85)	< 0.001
ECMO-related complications				
Intravascular hemolysis	29 (18)	9 (10)	20 (28)	0.004
Clogged circuit requiring change	17 (11)	3 (3)	14 (20)	< 0.001
Severe thrombocytopenia (< 50 G/L) ^a^	10 (6)	5 (6)	5 (7)	0.754
ECMO setting/insertion change(s) ^b^	8 (5)	4 (5)	4 (6)	0.051
Massive hemorrhage	70 (44)	34 (39)	36 (51)	0.127
Stroke				1.000
Ischemic	2 (1)	1 (1)	1 (1)	
Hemorrhagic	8 (5)	4 (5)	4 (6)	
Pulmonary embolism	23 (14)	16 (18)	7 (10)	0.138
Cardiac arrest	17 (11)	9 (10)	8 (11)	0.833
Pneumothorax	17 (11)	5 (6)	12 (17)	0.03
Antibiotic-treated VAP	159 (100)	75 (85)	63 (89)	0.516
≥ 1 treated bacteremia episode(s)	75 (47)	44 (51)	31 (44)	0.388

Values are expressed as median (interquartile range) or number (%)

*APRV* airway pressure release ventilation, *aPTT* activated partial thromboplastin time, ECMO extracorporeal membrane oxygenation, *ICU* intensive care unit, *Fr* French, *FiO*_*2*_ the fraction of inspired oxygen, *PEEP* positive end-expiratory pressure, *PaO*_*2*_ partial pressure of arterial oxygen, *PaCO*_*2*_ partial pressure of arterial carbon dioxide, *PAPV* positive airway-pressure ventilation, *PBW* predicted body weight, and *SOFA* Sequential Organ-Function Assessment, *VA* venoarterial, *VV* venovenous, *VAP* ventilator-associated pneumonia

^a^During the first 3 days

^b^Included ECMO-cannulation switches from VA to VV; VA to venous–arteriovenous (V-AV); and VV to V-AV

### ICU and ECMO-related complications

Despite significantly lower aPTT ratios during the first days on-ECMO after July 1, massive hemorrhage, hemorrhagic stroke and pulmonary embolism rates did not differ between periods. However, intravascular hemolysis and clogged circuits were more frequent after July 1. Pneumothorax occurred significantly more frequently during the second period (6% vs. 17%, *p* = 0.03). The frequencies of antibiotic-treated ventilator-associated pneumonia and bacteremia episodes remained very high and comparable between study periods. More than a third of our patients required renal replacement therapy while on-ECMO.

### Patient outcomes

**Fig. 1:**
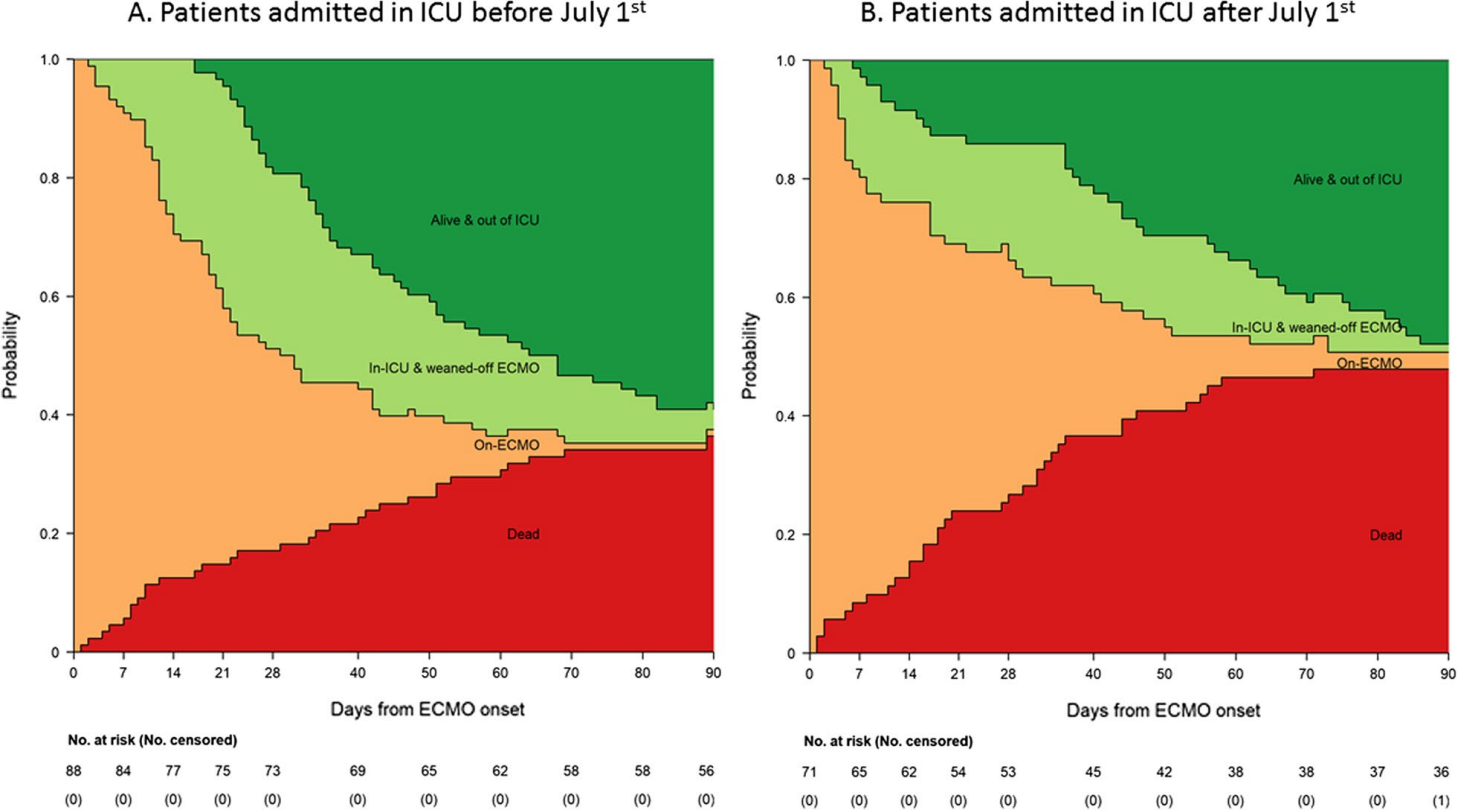
Multistate-model stacked probability plots for ICU patients admitted a before or b after July 1. The plots illustrate the probability of being in each endpoint state—On-ECMO, In-ICU and weaned-off ECMO, Alive and out of ICU or dead—over the 90 days post-ECMO implantation. The respective probabilities and mean lengths of stay (with 95% confidence intervals) for each of these four states are reported in Additional file 2. *ECMO* extracorporeal membrane oxygenation, *ICU* intensive care unit

**Fig. 2:**
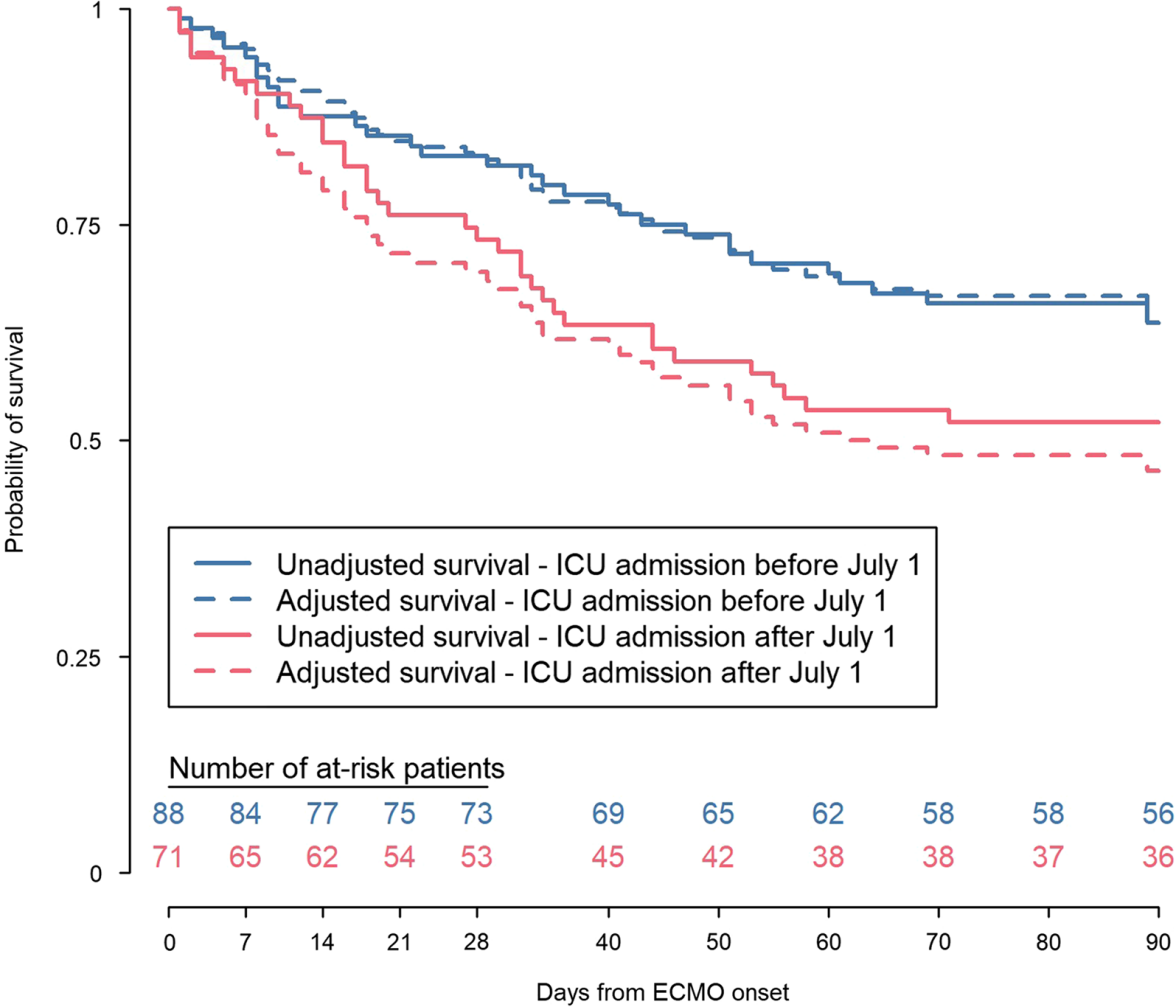
90-day Kaplan–Meier survival estimates post-ICU admission according to admission period. Adjusted (HR 2.27, 95% CI 1.02–5.07; *p* = 0.05) and unadjusted (log-rank test *p* = 0.108) survival-model values

On April 28, 2021, complete 90-day follow-up was obtained for all patients. The estimated state-occupation probabilities (95% CI) of being on-ECMO, in-ICU & weaned-off ECMO, alive and out of ICU or dead 90 days post-ECMO initiation, respectively, were: 1% (0.2–8%), 3% (1–10%), 59% (49–69%) and 36% (27–47%) during the first period, and 3% (0.7–11%), 1% (0.2–10%), 48% (37–60%) and 48% (37–60%) for patients admitted after July 1 (Fig. 1 and Additional file 4). Kaplan–Meier estimates of 90-day survival were 64% and 52%, respectively, for the first and the second periods (log-rank test *p* = 0.108) (Fig. 2). After adjusting for confounders, patients treated after July 1 had a significantly higher probability of death by day 90 (HR 2.27, 95% CI 1.02–5.07).

Median (IQR) ECMO durations during the first and second periods, respectively, were 19 (10–35) and 18 (5–35) days (*p* = 0.949), and 22 (12–48) and 33 (8–62) days among 90-day survivors (*p* = 0.466). Similarly, ICU and hospital lengths of stay did not differ between periods (Additional file 5).

### Predictors of 90-day mortality

The multivariable Cox regression model identified the following patient characteristics as being significantly associated with higher 90-day mortality: being admitted to the ICU after July 1, older age and pre-ECMO SOFA cardiovascular component score ≥ 3 (Table 3). In addition, a higher pre-ECMO PCO_2_ and longer ICU-admission-to-ECMO interval tended to be associated with higher 90-day mortality. Conversely, having received dexamethasone before ECMO implantation was a protective factor, whereas the SOFA renal component score, pre-ECMO driving pressure and PaO_2_/FiO_2_ ratio were not independently associated with higher risk of mortality. Multiple imputations of missing data yielded similar results but ICU admission after July 1 and dexamethasone treatment no longer reached statistical significance (Additional file 6).

**Table 3:** Factors at ECMO onset associated with 90-day mortality of severe COVID-19–ARDS patients on ECMO

Factor	Univariate HR (95% CI)	*p*	Multivariate HR (95% CI) ^a^	*p*
Date of ICU admission		0.110		0.050
Before July 1	–		–	
After July 1	1.47 (0.92–2.37)		2.27 (1.02–5.07)	
Age, years		< 0.001		0.006
˂45	–		–	
45–55	2.81 (1.26–6.29)		1.86 (0.74–4.65)	
˃55	5.13 (2.39–11.00)		3.55 (1.48–8.50)	
Days from ICU admission to ECMO		0.017		0.066
< 4 days	–		–	
4–7 days	1.37 (0.69–2.74)		2.51 (1.04–6.10)	
8–10 days	1.50 (0.68–3.29)		2.83 (1.04–7.70)	
> 10 days	2.82 (1.43–5.57)		3.02 (1.15–7.92)	
Driving pressure pre-ECMO, cmH_2_O		0.150		0.264
< 17	–		–	
17–20	0.95 (0.45–1.99)		0.84 (0.37–1.94)	
20–22	1.18 (0.57–2.42)		1.04 (0.47–2.29)	
˃22	1.98 (1.03–3.81)		1.85 (0.86–3.98)	
PaO_2_/FiO_2_ pre-ECMO		0.621		0.815
< 53	–		–	
53–61	0.80 (0.42–1.53)		1.08 (0.49–2.39)	
61–69	0.82 (0.42–1.61)		0.77 (0.33–1.81)	
˃69	1.19 (0.62–2.27)		1.12 (0.48–2.62)	
PaCO_2_ pre-ECMO, mmHg		0.007		0.066
< 0	–		–	
50–57	0.72 (0.31–1.67)		0.79 (0.30–2.13)	
57–66	1.64 (0.82–3.30)		2.10 (0.91–4.86)	
˃66	2.35 (1.20–4.59)		2.11 (0.98–4.53)	
Dexamethasone started pre-ECMO	1.07 (0.67–1.71)	0.783	0.37 (0.17–0.79)	0.012
SOFA score CV component ≥ 3	1.82 (1.13–2.94)	0.013	2.44 (1.31–4.55)	0.004
SOFA score renal component ≥ 3	1.58 (0.87–2.89)	0.156	1.50 (0.72–3.13)	0.296

*CV* cardiovascular, *SOFA* sequential organ failure assessment, *ICU* intensive care unit, *HR* hazard ratio, *CI* confidence interval

^a^Complete analysis of 55 patients

## Discussion

Patient mortality 90 days after starting ECMO support in our experienced center for the most severe forms of COVID-19 ARDS increased from 36% before to 48% after July 1, 2020. Patients admitted after that date were older, had longer ICU-admission-to-intubation intervals, with more frequent HFNO or NIV use, and most of them had received dexamethasone at ECMO onset. Independent pre-ECMO predictors of 90-day mortality for the entire cohort were older age, longer ICU-admission-to-ECMO interval, cardiovascular dysfunction, not having received dexamethasone and being admitted after July 1, 2020.

The higher mortality rate of our ECMO-treated COVID-19 patients admitted after July 1, 2020, was unexpected. Differences in patient characteristics, management and SARS-CoV-2 pathogenicity might explain that observation. First, the responsibility of pathogenicity seems unlikely, since the original European SARS-CoV-2 represented > 90% of strains circulating in France until mi-January 2021, after which the B.1.1.7 (Alpha) variant progressively became dominant. Second, most patient characteristics were comparable for the two periods, except for a 5-year higher median age during the second period. However, pre-ECMO patient management differed markedly after July 1, 2020. After RECOVERY trial results (published in June 2020) demonstrated lower mortality of patients randomized to receive 10 days of dexamethasone (6 mg/day) compared to usual care of mechanically ventilated COVID-19 patients (29% vs. 41%, respectively), most patients received corticosteroids. Pertinently, having started dexamethasone pre-ECMO was significantly associated with lower 90-day mortality in our predictive model. Also, more frequent use of dexamethasone and high-dose corticosteroids for persistent ARDS after July 1 was not associated with a higher rate of infectious complications.

The other notable patient-management differences were more frequent HFNO or NIV use and for longer durations before intubation during the second period. Although noninvasive respiratory support was shown to lower the need for intubation and invasive mechanical ventilation, COVID-19 patients for whom this strategy failed may have been at increased risk of morbidity and mortality [24, 25]. Indeed, strong and dysregulated spontaneous respiratory efforts, associated with wide transpulmonary pressure swings might heighten the risk of harmful “self-inflicted lung injury” on HFNO or NIV, with more frequent fibrotic evolution of COVID-19 pneumonia [26, 27]. That phenomenon could even be more difficult to detect in COVID-19 patients, who frequently experience dissociation between profound hypoxemic respiratory failure, and only moderate feelings and signs of respiratory distress and dyspnea [28]. Pertinently, the higher pneumothorax rate after July 1, despite similar ventilatory management during both periods, supports that hypothesis, even though pre-ECMO respiratory mechanic parameters did not differ significantly between periods. It should also be noted that although on-ECMO prone-positioning remained frequent (> 80%) after July 1, fewer patients received continuous neuromuscular blockade. Lastly, pulmonary embolism, stroke and hemorrhage rates did not change, while intravascular hemolysis and clogged circuits requiring change were more frequent after July 1. Those observations might reflect the significantly lower anticoagulation delivered to our second-period patients.

We acknowledge several limitations of this study. Our patients were treated in a high-volume, experienced ECMO center. Because better post-ECMO outcomes have been reported in such centers [4, 29], caution is required when extrapolating these results to less-experienced ECMO centers. Second, our study took place before SARS-CoV-2 variants associated with more severe ARDS forms became more prevalent (UK 20I/501Y.V1, South African 20H/501Y.V2, BR-P1 Brazilian, and Delta variants). ECMO outcomes of patients infected with those more virulent strains should be evaluated urgently. Third, COVID-19 management evolved throughout the study period, with widespread use of corticosteroids and interleukin-6 inhibitors. Therefore, patients refractory to several COVID-19 treatments who received ECMO during the second period might be considered sicker than those managed before July 1, 2020. Moreover, we cannot exclude that some residual confounding factors may not have been taken into account in our logistic model. Lastly, our cohort’s mortality rate could evolve further, as some patients were still hospitalized 90 days after ICU admission.

## Conclusion

In conclusion, survival of ECMO-rescued patients with severe COVID-19 ARDS in our experienced center has declined over time and is no longer comparable to that of non-COVID ECMO patients. Failure of prolonged non-invasive oxygenation strategies before intubation and increased lung damage, as well as selection of patients already refractory to specific COVID-19 treatments might partially explain this outcome. Although other residual confounding factors may not have been considered herein, the duration of noninvasive respiratory support, e.g., continuous positive airway pressure, HFNO or NIV, should perhaps be integrated in the decision to initiate ECMO for severe COVID-19 patients.

## Supplementary Information

**Additional file 1**. All possible transition probabilities from one state to another over time.

**Additional file 2**. The estimated probabilities (95% CI) of all possible transitions from one state to another for (A) 88 patients admitted to the ICU before July 1, 2020, and (B) 71 patients admitted to the ICU after July 1, 2020.

**Additional file 3**. Additional information concerning patients’ characteristics and complications on-ECMO according to ICU-admission period.

**Additional file 4**. Probabilities of being in each of the four endpoint states and the mean time spent in each one on days 28, 40, 50, 60, and 90 post-ECMO onset according to the ICU-admission date.

**Additional file 5**. ECMO duration, and ICU and hospital lengths of stay according to the ICU-admission period.

**Additional file 6**. Predictive factors associated with 90-day mortality of 159 adults with COVID-19 severe ARDS with multiple imputations.

## Data Availability

The datasets used during the current study are available from the corresponding author on reasonable request.

## Abbreviations

aPTT: Activated partial thromboplastin time
ARDS: Acute respiratory distress syndrome
ECMO: Extracorporeal membrane oxygenation
VA/VV-ECMO: Venoarterial/venovenous-extracorporeal membrane oxygenation
ICU: Intensive care unit
COVID-19: Coronavirus disease 2019
HFNO: High-flow oxygen through a nasal cannula
NIV: Noninvasive ventilation
SAPS: Simplified Acute Physiology Score
SARS-CoV-2: Severe acute respiratory syndrome coronavirus-2
RESP: Respiratory extracorporeal membrane oxygenation survival prediction
SOFA: Sequential organ-failure assessment
