# Supplementary material for: Evolving outcomes of extracorporeal membrane oxygenation support for severe COVID-19 ARDS in Sorbonne hospitals, Paris
Source: Crit Care. 2021 Oct 9;25:355. doi: 10.1186/s13054-021-03780-6 (PMC8502094; doi:10.1186/s13054-021-03780-6)
Supplement: Supplementary file 1 — Additional file 1. All possible transition probabilities from one state to another over time. [file 13054_2021_3780_MOESM1_ESM.docx]

**eFile 1 All possible transition probabilities from one state to another over time.**


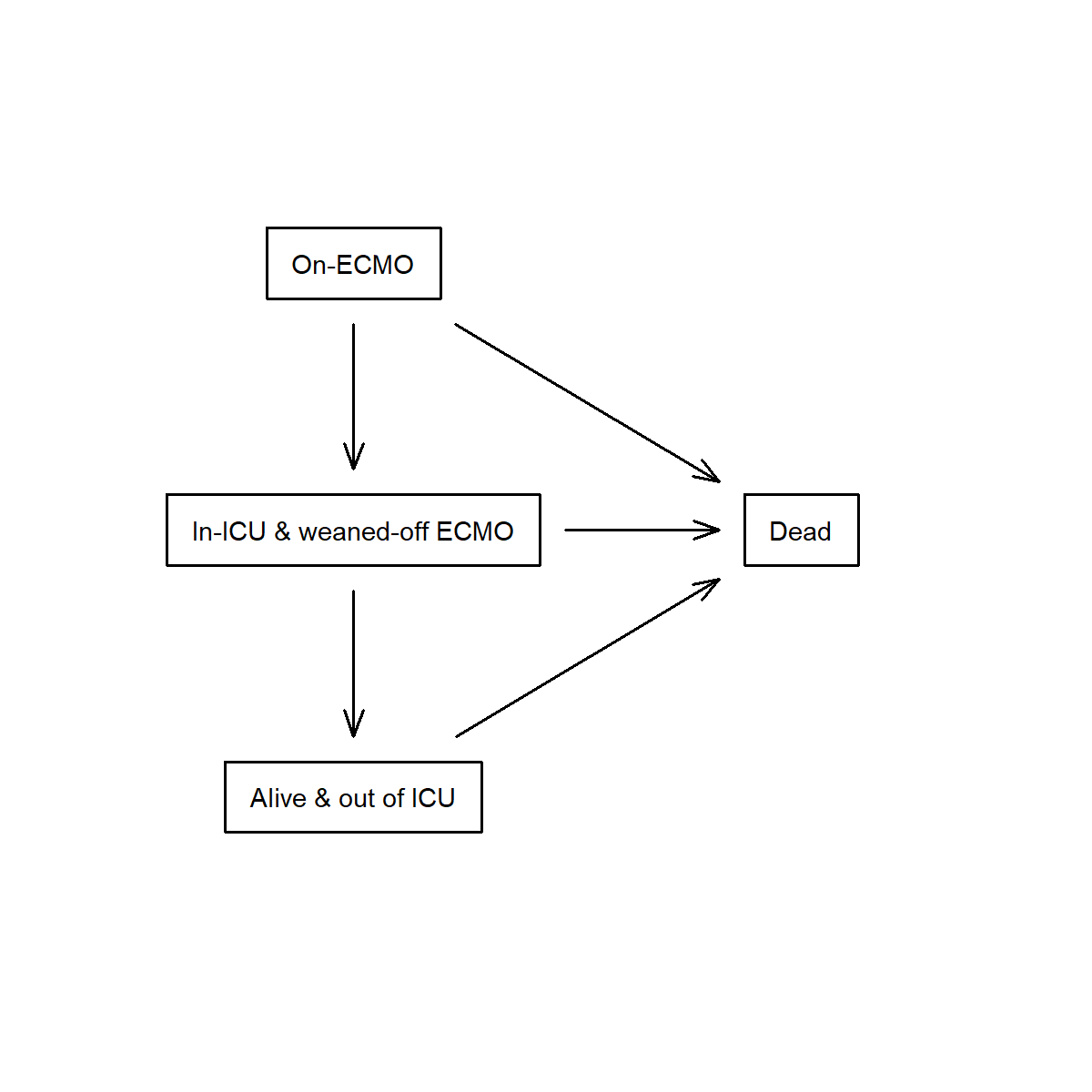


*ECMO, extracorporeal membrane oxygenation; ICU, intensive care unit*
