# Supplementary material for: Evolving outcomes of extracorporeal membrane oxygenation support for severe COVID-19 ARDS in Sorbonne hospitals, Paris
Source: Crit Care. 2021 Oct 9;25:355. doi: 10.1186/s13054-021-03780-6 (PMC8502094; doi:10.1186/s13054-021-03780-6)
Supplement: Supplementary file 2 — Additional file 2. The estimated probabilities (95% CI) of all possible transitions from one state to another for (A) 88 patients admitted to the ICU before July 1, 2020, and (B) 71 patients admitted to the ICU after July 1, 2020. [file 13054_2021_3780_MOESM2_ESM.docx]

**eFile 2 The estimated probabilities (95% CI) of all possible transitions from one state to another for (A) 88 patients admitted to the ICU before July 1, 2020, and (B) 71 patients admitted to the ICU after July 1, 2020.**
