# Supplementary material for: Evolving outcomes of extracorporeal membrane oxygenation support for severe COVID-19 ARDS in Sorbonne hospitals, Paris
Source: Crit Care. 2021 Oct 9;25:355. doi: 10.1186/s13054-021-03780-6 (PMC8502094; doi:10.1186/s13054-021-03780-6)
Supplement: Supplementary file 3 — Additional file 3. Additional information concerning patients’ characteristics and complications on-ECMO according to ICU-admission period. [file 13054_2021_3780_MOESM3_ESM.docx]

**eFile 3: Additional information concerning patients’ characteristics and complications on-ECMO according to ICU-admission period.**

|  | **All**  **(N=159)** | **ICU admission before July 1^st^**  **(N=88)** | **ICU admission after July 1^st^ (N=71)** | **P value** |
| --- | --- | --- | --- | --- |
| Type of ECMO support |  |  |  | 0.846 |
| Femoral–jugular VV | 150 (94) | 81 (92) | 69 (97) |  |
| Femoral–femoral VV | 4 (3) | 3 (3) | 1 (1) |  |
| Femoral–femoral VA | 3 (2) | 2 (2) | 1 (1) |  |
| Femoral–jugular–femoral, VA-V | 1 (1) | 1 (1) | 0 (0) |  |
| ECMO blood flow L/min | 5.0 (4.5-5.5) | 5.0 (4.6-5.5) | 4.9 (4.4-5.2) | 0.053 |
| Sweep gas flow, L/min | 5 (4-5) | 5 (4-6) | 2 (2-4) | <0.001 |
| Membrane FmO_2_, % | 100 (100-100) | 100 (100-100) | 100 (100-100) | 0.375 |
| **ECMO Day-1** |  |  |  |  |
| Total SOFA score |  |  |  |  |
| Renal component ≥3 | 33 (21) | 20 (23) | 13 (18) | 0.692 |
| Cardiovascular component ≥3 | 80 (50) | 44 (50) | 36 (50) | 0.568 |
| Haematological component ≥3 | 6 (4) | 4 (5) | 2 (3) | 0.853 |
| Blood gases on ECMO |  |  |  |  |
| pH | 7.39 (7.32-7.46) | 7.40 (7.36-7.47) | 7.38 (7.30-7.45) | 0.04 |
| PaO_2_, mm Hg | 87 (73-117) | 82 (71-102) | 97 (75-142) | 0.003 |
| PaCO_2_, mm Hg | 46 (40-52) | 45 (40-50) | 46 (40-54) | 0.258 |
| Bicarbonate, mmol/L | 29 (24-31) | 29 (25-31) | 28 (23-31) | 0.191 |
| Arterial lactate, mmol/L | 1.7 (1.3-2.3) | 1.7 (1.4-2.1) | 1.8 (1.3-2.4) | 0.411 |
| Adjuvant therapy on ECMO day 1 |  |  |  |  |
| Neuromuscular blockade | 71 (45) | 70 (79) | 1 (1) | <0.001 |
| Prone positioning | 10 (6) | 8 (9) | 2 (3) | 0.187 |
| Inhaled nitric oxide | 3 (2) | 3 (4) | 0 (0) | 0.254 |
| Renal replacement therapy | 19 (12) | 12 (14) | 7 (10) | 0.465 |
| Pneumothorax | 5 (3) | 2 (2) | 3 (4) | 0.657 |
| Cardiac arrest | 3 (2) | 2 (2) | 1 (2) | 1.000 |
| **During the ECMO run** |  |  |  |  |
| SOFA score on ECMO-day 3 | 10 (8-14) | 11 (8-14) | 10 (8-13) | 0.336 |
| SOFA score on ECMO-day 7 | 12 (8-14) | 11 (8-13) | 12 (9-14) | 0.345 |
| Received COVID-19 specific treatment |  |  |  |  |
| Remdesivir | 17 (11) | 8 (9) | 9 (13) | 0.467 |
| Lopinavir/ritonavir | 20 (13) | 20 (23) | 0 (0) | <0.001 |
| Tocilizumab | 9 (6) | 8 (9) | 1 (1) | 0.042 |
| DXM ≤ 6mg during the first 15 days | 73 (46) | 13 (15) | 60 (85) | <0.001 |
| Other | 40 (25) | 33 (37) | 7 (10) | <0.001 |
| Included in a RCT on SARS-CoV-2 therapy | 13 (9) | 13 (15) | 0 (0) | 0.002 |
| Massive haemorrhage | 70 (44) | 34 (39) | 36 (51) | 0.127 |
| Oronasal bleeding | 47 (30) | 21 (24) | 26 (37) | 0.080 |
| Haemothorax | 12 (8) | 8 (9) | 4 (6) | 0.412 |
| Cannula | 16 (10) | 5 (6) | 11 (15) | 0.041 |
| Other site | 21 (13) | 12 (14) | 9 (13) | 0.859 |
| Blood-product transfusion |  |  |  |  |
| Patients who received ≥1 red-cell units | 111 (71) | 66 (75) | 45 (66) | 0.228 |
| Number of red-cell units/patient | 3 (0-9) | 3 (1-11) | 2 (0-8) | 0.214 |
| Patients who received ≥1 platelet units | 25 (16) | 12 (14) | 13 (19) | 0.355 |
| Patients who received ≥1 FFP units | 19 (12) | 10 (11) | 9 (13) | 0.723 |

*Values are expressed as median (interquartile range) or number (%).*

*APRV denotes airway pressure release ventilation; aPTT activated partial thromboplastin time, ECMO extracorporeal membrane oxygenation, ICU intensive care unit, FFP, fresh frzen plasma, FiO_2_ the fraction of inspired oxygen, RCT, randomised controlled trial, SOFA Sequential Organ-Function Assessment, VA venoarterial, VV venovenous, VAP ventilator associated pneumonia*
