# Supplementary material for: Evolving outcomes of extracorporeal membrane oxygenation support for severe COVID-19 ARDS in Sorbonne hospitals, Paris
Source: Crit Care. 2021 Oct 9;25:355. doi: 10.1186/s13054-021-03780-6 (PMC8502094; doi:10.1186/s13054-021-03780-6)
Supplement: Supplementary file 4 — Additional file 4. Probabilities of being in each of the four endpoint states and the mean time spent in each one on days 28, 40, 50, 60, and 90 post-ECMO onset according to the ICU-admission date. [file 13054_2021_3780_MOESM4_ESM.docx]

**eFile 4: Probabilities of being in each of the four endpoint states and the mean time spent in each one on days 28, 40, 50, 60, and 90 post-ECMO onset according to the ICU-admission date.**

|  | **ICU admission before July 1^st^**  **(N=88)** | | **ICU admission after July 1^st^**  **(N=71)** | |
| --- | --- | --- | --- | --- |
| **Day and Endpoint State** | **% (95% CI) ^a^** | **Mean Days in Each State (95% CI) ^b^** | **% (95% CI) ^a^** | **Mean Days in Each State (95% CI) ^b^** |
| Day 28 |  |  |  |  |
| On-ECMO | 0.34 (0.25-0.45) | 18.1 (16.2-20.0) | 0.39 (0.29-0.52) | 17.6 (15.1-20.1) |
| In-ICU & weaned-off ECMO | 0.29 (0.21-0.40) | 7.0 (4.5-7.6) | 0.20 (0.12-0.31) | 4.1 (2.5-5.9) |
| Alive & out of ICU | 0.19 (0.12-0.29) | 0.9 (0.4-1.4) | 0.14 (0.08-0.25) | 2.2 (0.9-3.7) |
| Died | 0.17 (0.11-0.27) | 3.9 (1.6-4.4) | 0.27 (0.18-0.39) | 4.1 (2.4-6.1) |
| Day 40 |  |  |  |  |
| On-ECMO | 0.22 (0.14-0.32) | 21.4 (18.6-24.2) | 0.24 (0.16-0.36) | 21.2 (17.8-24.8) |
| In-ICU & weaned-off ECMO | 0.23 (0.15-0.33) | 9.3 (7.2-11.5) | 0.17 (0.10-0.28) | 6.6 (4.2-9.2) |
| Alive & out of ICU | 0.33 (0.24-0.43) | 3.9 (2.6-5.4) | 0.22 (0.14-0.34) | 4.1 (2.0-6.7) |
| Died | 0.23 (0.15-0.33) | 5.4 (3.2-7.6) | 0.37 (0.27-0.49) | 8.0 (5.3-11.0) |
| Day 50 |  |  |  |  |
| On-ECMO | 0.14 (0.08-0.23) | 23.0 (19.7-24.2) | 0.14 (0.08-0.25) | 23.2 (19.3-27.3) |
| In-ICU & weaned-off ECMO | 0.19 (0.12-0.29) | 11.5 (9.1-14.2) | 0.16 (0.09-0.26) | 8.1 (5.3-11.1) |
| Alive & out of ICU | 0.41 (0.31-0.52) | 7.6 (5.4-10.0) | 0.30 (0.20-0.42) | 6.8 (4.0-10.2) |
| Died | 0.26 (0.18-0.37) | 7.8 (4.9-10.9) | 0.41 (0.30-0.59) | 11.9 (8.2-15.8) |
| Day 60 |  |  |  |  |
| On-ECMO | 0.06 (0.02-0.13) | 23.9 (20.3-27.4) | 0.07 (0.03-0.16) | 24.2 (19.9-28.9) |
| In-ICU & weaned-off ECMO | 0.17 (0.11-0.27) | 13.2 (10.5-16.4) | 0.13 (0.07-0.23) | 9.7 (6.6-13.1) |
| Alive & out of ICU | 0.47 (0.37-0.57) | 12.1 (9.0-15.4) | 0.34 (0.24-0.46) | 9.8 (6.0-14.1) |
| Died | 0.31 (0.22-0.41) | 10.7 (7.0-14.5) | 0.46 (0.36-0.59) | 16.2 (11.5-21.0) |
| Day 90 |  |  |  |  |
| On-ECMO | 0.01 (0.002-0.08) | 24.6 (20.6-28.5) | 0.03 (0.007-0.11) | 25.2 (20.4-30.6) |
| In-ICU & weaned-off ECMO | 0.03 (0.01-0.10) | 16.2 (12.9-20.0) | 0.01 (0.002-0.10) | 12.9 (8.3-15.5) |
| Alive & out of ICU | 0.59 (0.49-0.69) | 28.4 (22.8-34.7) | 0.48 (0.37-0.60) | 22.3 (16.0-29.0) |
| Died | 0.36 (0.27-0.47) | 20.8 (14.7-27.1) | 0.48 (0.37-0.60) | 30.5 (22.9-38.2) |

*^a^ Probability of being in each endpoint state at the defined day after ECMO initiation.*

*^b^ Mean number of days spent in each endpoint state on the indicated day after ECMO initiation.*

*ECMO, extracorporeal membrane oxygenation, ICU intensive care unit, CI confidence interval.*
