# Supplementary material for: Evolving outcomes of extracorporeal membrane oxygenation support for severe COVID-19 ARDS in Sorbonne hospitals, Paris
Source: Crit Care. 2021 Oct 9;25:355. doi: 10.1186/s13054-021-03780-6 (PMC8502094; doi:10.1186/s13054-021-03780-6)
Supplement: Supplementary file 5 — Additional file 5. ECMO duration, and ICU and hospital lengths of stay according to the ICU-admission period. [file 13054_2021_3780_MOESM5_ESM.docx]

**eFile 5: ECMO duration, and ICU and hospital lengths of stay according to the ICU-admission period.**

|  | **ICU admission before July 1^st^**  **(N=88)** | **ICU admission after July 1^st^ (N=71)** | ***P* value** |
| --- | --- | --- | --- |
| ECMO duration | 19 (10-35) | 18 (5-35) | 0.949 |
| ECMO duration in survivors | 22 (12-48) | 33 (8-62) | 0.466 |
| ICU LOS, days | 36 (23-56) | 34 (14-55) | 0.269 |
| ICU LOS in survivors | 50 (32-75) | 56 (36-75) | 0.949 |
| Hospital LOS, days | 52 (36-84) | 34 (18-67) | 0.062 |
| Hospital LOS in survivors | 76 (51-NA) | 74 (49-154) | 0.574 |
