# Supplementary material for: Evolving outcomes of extracorporeal membrane oxygenation support for severe COVID-19 ARDS in Sorbonne hospitals, Paris
Source: Crit Care. 2021 Oct 9;25:355. doi: 10.1186/s13054-021-03780-6 (PMC8502094; doi:10.1186/s13054-021-03780-6)
Supplement: Supplementary file 6 — Additional file 6. Predictive factors associated with 90-day mortality of 159 adults with COVID-19 severe ARDS with multiple imputations. [file 13054_2021_3780_MOESM6_ESM.docx]

**eFile 6: Predictive factors associated with 90-day mortality of 159 adults with COVID-19 severe ARDS with multiple imputations.**

|  | **Multivariate HR**  **(95% CI)** | ***P* value** |
| --- | --- | --- |
| Date of ICU admission |  |  |
| After July, 1^st^ | 1.79 (0.83 - 3.87) | 0.133 |
| Age, years |  |  |
| ˂ 45 | – |  |
| 45-55 | 2.44 (1.03 - 5.80) | 0.044 |
| ˃ 55 | 4.21 (1.83 – 9.65) | 0.001 |
| Time from ICU admission to ECMO, days |  |  |
| < 4 days | – |  |
| 4 to 7 days | 2.30 (1.07 - 4.95) | 0.034 |
| 8 to 10 days | 1.53 (0.63 - 3.73) | 0.338 |
| > 10 days | 3.02 (1.03 - 5.17) | 0.043 |
| Driving pressure before ECMO, cmH_2_O |  |  |
| < 17 | – |  |
| 17-20 | 0.82 (0.36-1.88) | 0.633 |
| 20-22 | 1.06 (0.47-2.40) | 0.883 |
| ˃ 22 | 1.78 (0.86-3.67) | 0.117 |
| PaO_2_/FiO_2_ before ECMO |  |  |
| < 53 | – |  |
| 53-61 | 0.80 (0.37 - 1.69) | 0.543 |
| 61-69 | 0.68 (0.31 - 1.47) | 0.317 |
| ˃ 69 | 1.27 (0.61 - 2.69) | 0.516 |
| PaCO_2_ before ECMO, mm Hg |  |  |
| < 50 | – |  |
| 50-57 | 0.94 (0.37 - 2.35) | 0.889 |
| 57-66 | 2.04 (0.83 - 4.98) | 0.114 |
| ˃ 66 | 2.18 (1.04 - 4.58) | 0.040 |
| Dexamethasone initiated before ECMO | 0.56 (0.27 - 1.14) | 0.107 |
| ***During the first 24-hours in the ICU*** |  |  |
| CV component of the SOFA score ≥ 3 | 2.03 (1.15 - 3.60) | 0.016 |
| Renal component of the SOFA score ≥ 3 | 1.02 (0.50 - 2.10) | 0.949 |

*CV, cardiovascular; SOFA, Sequential Organ Failure Assessment; ICU, intensive care unit; HR, hazard ratio; CI, confidence interval.*
